# Spatiotemporal distribution and bionomics of *Anopheles stephensi* in different eco-epidemiological settings in Ethiopia

**DOI:** 10.1186/s13071-024-06243-3

**Published:** 2024-03-31

**Authors:** Temesgen Ashine, Adane Eyasu, Yehenew Asmamaw, Eba Simma, Endalew Zemene, Adrienne Epstein, Rebecca Brown, Nigatu Negash, Abena Kochora, Alison M. Reynolds, Mikiyas Gebremichael Bulto, Temesgen Tafesse, Alemayehu Dagne, Biniyam Lukus, Endashaw Esayas, Sinknesh Wolde Behaksra, Kidist Woldekidan, Fikregabrail Aberra Kassa, Jimma Dinsa Deressa, Muluken Assefa, Dereje Dillu, Gudissa Assefa, Hiwot Solomon, Ahmed Zeynudin, Fekadu Massebo, Luigi Sedda, Martin James Donnelly, Anne L. Wilson, David Weetman, Endalamaw Gadisa, Delenasaw Yewhalaw

**Affiliations:** 1 Department of Biology, College of Natural and Computational Sciences, Arba Minch University, Arba Minch, Ethiopia; 2 Malaria and NTD Research Division, Armauer Hansen Research Institute, Addis Ababa, Ethiopia; 3 Tropical and Infectious Diseases Research Center, Jimma University, Jimma, Ethiopia; 4 Department of Biology, College of Natural Sciences, Jimma University, Jimma, Ethiopia; 5 School of Medical Laboratory Sciences, Institute of Health, Jimma University, Jimma, Ethiopia; 6 Department of Vector Biology, Liverpool School of Tropical Medicine, Pembroke Place, Liverpool, L3 5QA UK; 7 Disease Prevention and Control Directorate, Ethiopian Federal Ministry of Health, Addis Ababa, Ethiopia; 8 Lancaster Ecology and Epidemiology Group, Lancaster Medical School, Lancaster University, Lancaster, UK

**Keywords:** *Anopheles stephensi*, Spatiotemporal distribution, Blood meal source, Sporozoite rate, Household’s exposure, Ethiopia

## Abstract

**Background:**

Malaria is a major public health concern in Ethiopia, and its incidence could worsen with the spread of the invasive mosquito species* Anopheles stephensi* in the country. This study aimed to provide updates on the distribution of *An. stephensi* and likely household exposure in Ethiopia.

**Methods:**

Entomological surveillance was performed in 26 urban settings in Ethiopia from 2021 to 2023. A kilometer-by-kilometer quadrant was established per town, and approximately 20 structures per quadrant were surveyed every 3 months. Additional extensive sampling was conducted in 50 randomly selected structures in four urban centers in 2022 and 2023 to assess households’ exposure to *An. stephensi*. Prokopack aspirators and CDC light traps were used to collect adult mosquitoes, and standard dippers were used to collect immature stages. The collected mosquitoes were identified to species level by morphological keys and molecular methods. PCR assays were used to assess *Plasmodium* infection and mosquito blood meal source.

**Results:**

Catches of adult *An. stephensi* were generally low (mean: 0.15 per trap), with eight positive sites among the 26 surveyed. This mosquito species was reported for the first time in Assosa, western Ethiopia. *Anopheles stephensi* was the predominant species in four of the eight positive sites, accounting for 75–100% relative abundance of the adult *Anopheles* catches. Household-level exposure, defined as the percentage of households with a peridomestic presence of *An. stephensi*, ranged from 18% in Metehara to 30% in Danan. *Anopheles arabiensis* was the predominant species in 20 of the 26 sites, accounting for 42.9–100% of the *Anopheles* catches. Bovine blood index, ovine blood index and human blood index values were 69.2%, 32.3% and 24.6%, respectively, for *An. stephensi*, and 65.4%, 46.7% and 35.8%, respectively, for *An. arabiensis*. None of the 197 *An. stephensi* mosquitoes assayed tested positive for *Plasmodium* sporozoite, while of the 1434 *An. arabiensis* mosquitoes assayed, 62 were positive for *Plasmodium* (10 for *P. falciparum* and 52 for *P. vivax*).

**Conclusions:**

This study shows that the geographical range of *An. stephensi* has expanded to western Ethiopia. Strongly zoophagic behavior coupled with low adult catches might explain the absence of *Plasmodium* infection. The level of household exposure to *An. stephensi* in this study varied across positive sites. Further research is needed to better understand the bionomics and contribution of *An. stephensi* to malaria transmission.

**Graphical Abstract:**

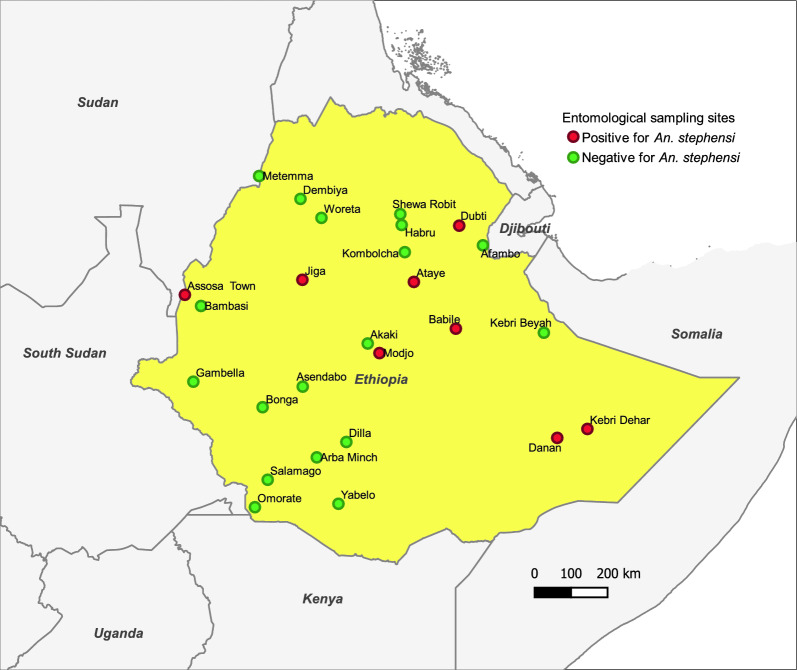

**Supplementary Information:**

The online version contains supplementary material available at 10.1186/s13071-024-06243-3.

## Background

Malaria remains a threat to global public health, with 249 million cases and 608,000 deaths in 2022 [1]. The WHO African region is disproportionately affected, and approximately 78% of malaria-related deaths in the region were in children aged < 5 years [1–4]. More than half of all Ethiopians, mainly those in rural areas, are at risk of contracting malaria [5–7]. Unlike most African countries, clinical malaria of public health importance in Ethiopia is caused by both *Plasmodium falciparum* (*P. falciparum*) and *P. vivax*, which co-occur in all malarious areas, with the prevalence attributable to each parasite dependent on ecological settings and seasons [7, 8]. The transmission of malaria is highly variable due to the diverse eco-topography and climate conditions and, in general, transmission is bimodal, mainly occurring following the rainy seasons, “*Kiremt” and* “*Belg,”* which are associated with major (long) and minor (short) transmission periods, respectively [8]. Since the 2000s, comprehensive preventative and case management interventions, including improved coverage of long-lasting insecticide-treated bed-nets and indoor residual spraying, the rollout and scale-up of artemisinin-based combination therapy, the deployment of a more sensitive and specific rapid diagnostic test (histidine-rich protein-2/3 [HRP2/3]-based test) and treatment at the grassroots level through health extension programs have achieved successive reductions in malaria burden [7, 9–11]. As a consequence, the aim of Ethiopia is to achieve zero indigenous malaria cases by 2030 [8, 12]. However, the country has experienced a nationwide resurgence in recent years and an unprecedented increase in case burden [1, 13]. Possible contributing factors include insecticide resistance in the primary malaria vector, the COVID-19 pandemic, the emergence of the HRP2/3 deletion, deterioration of the healthcare system, internal conflicts and invasion by the exotic malaria vector, *Anopheles stephensi* [14–17].

Until recently, about 46 *Anopheles* species and subspecies were recorded in Ethiopia [18, 19]. However, only a few *Anopheles* species, including *An*. *arabiensis, An. pharoensis, An. funestus* and *An. nili*, were incriminated as vectors of malaria [20]. *Anopheles arabiensis* is the primary malaria vector in most malaria-endemic areas [21–23]. However, its abundance, host preference and *Plasmodium* sporozoite rate vary across ecological gradients and epidemiological settings [22–25]. *Anopheles pharoensis* is of secondary importance [20, 23], with *An. funestus* and *An. nili* playing lesser roles in malaria transmission [22, 23].

*Anopheles stephensi* is an efficient urban malaria vector in southeast Asia and the Gulf Region [26] but is currently expanding its geographical range in Africa, where there have been reports of its presence in Djibouti [27], Ethiopia [17], Sudan [28], Somalia [29] and, more recently, Nigeria, Eritrea, Ghana and Kenya [30]. *Anopheles stephensi* is known to readily invade urban environments, and immature stages thrive in artificial aquatic habitats, with the consequent potential to increase malaria incidence in cities [27, 31] or reintroduce the disease into regions where it has been successfully eliminated [32–35].

In Ethiopia, since the first detection of *An. stephensi* in Kebri Dehar, Somali region [17], surveillance has confirmed its presence in the central, northeast, northwest and southwest parts of the country [36, 37]. Recent studies have shown that *An. stephensi* is a permissive host to *P. falciparum* and *P. vivax* infection [16, 37]. The results of a study from Dire Dawa City, eastern Ethiopia, suggest that *An. stephensi* was responsible, at least in part, for a malaria outbreak [38]. Similarly, in Djibouti, an upsurge in malaria incidence was observed following the detection of *An. stephensi,* providing further evidence for the potential for increased risk [39, 40]. In line with the WHO’s call for strengthened entomological surveillance of *An. stephensi* [41], this study aims to update current data on the distribution of *An. stephensi* across Ethiopia and to increase understanding of the patterns of household exposure to *An. stephensi* across the country.

## Methods

### Study area

**Fig. 1:**
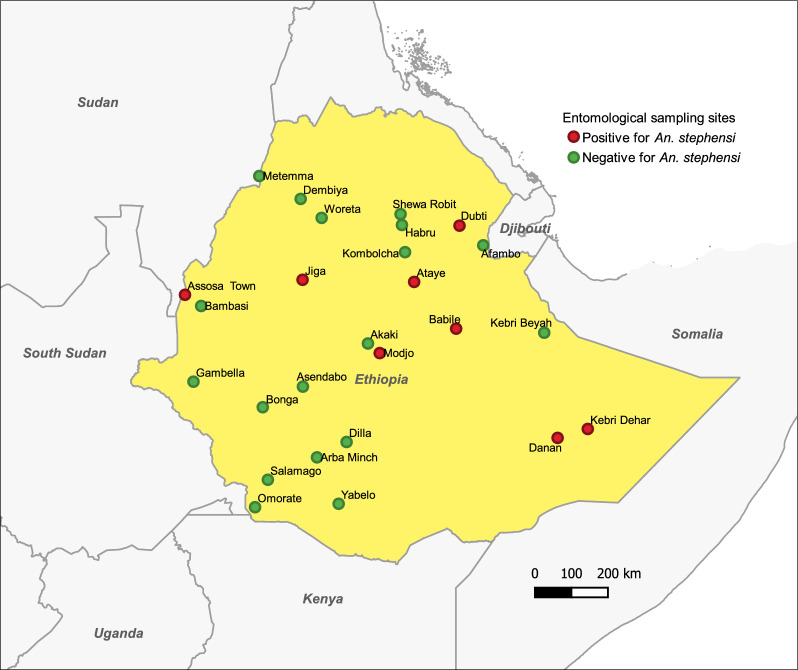
Map of study urban centers, with the colored dots indicating *Anopheles stephensi*-positive (red) and -negative sites (green)

Twenty-six urban centers were selected for this study (Fig. 1). A range of variables, including ecological setting, presence of dry ports and major transportation corridors, *An. stephensi* habitat suitability modeling and malaria endemicity, were considered when selecting the sites [36, 42]. The study sites (Additional file 1: Table S1) are located at altitudes between 339 and 2355 m a.s.l. and range from hot-desert lowland to humid highland environments. The mean annual temperature ranges from 30.9 °C in Afambo, the northeastern tip of the country, to 15.6 °C in Akaki, central Ethiopia, and the mean annual rainfall ranges from 224 to 1883 mm^2^ [43].

### Study design

#### Spatiotemporal distribution of *An. stephensi*

**Fig. 2:**
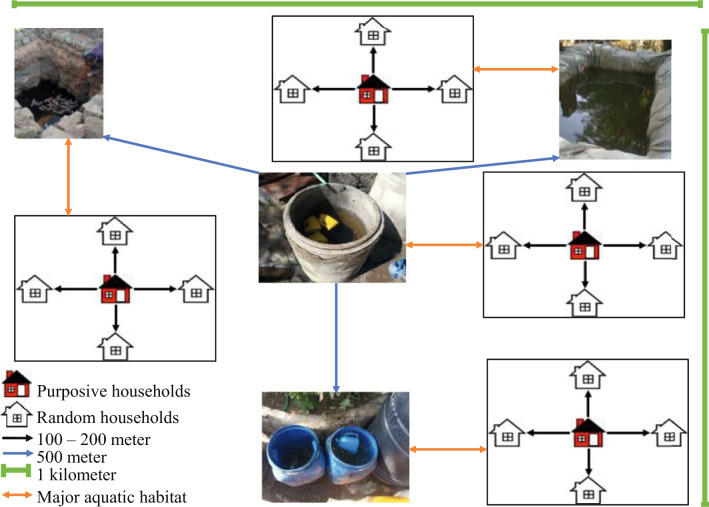
Schematic showing the approach used to select habitats and households for *Anopheles* mosquito collection, with the aim to study the spatiotemporal distribution of *An. stephensi* in Ethiopia

Four rounds of adult and immature stage collections were conducted at approximately 3-month intervals from November 2021 to January 2023. At each of the 26 study sites, a preliminary survey was first conducted to locate potential aquatic habitats of *Anopheles* mosquitoes outside compound/property limit of human dwellings/households. Based on the availability of aquatic habitats, we delineated a 1- × 1-km quadrant for entomological sampling (Fig. 2). In each quadrant, 20 households were selected for adult mosquito collection, of which four households were purposively selected based on their proximity to a major aquatic habitat and four were randomly selected in different directions from each of the four purposively chosen dwellings. Immature-stage mosquitoes were collected from all potential aquatic habitats within the compounds/property limits of the households selected for adult collection as well as from the purposively identified aquatic habitats within the quadrant beyond the compounds/property limits of the selected dwellings. Catches from the immature-stage collections were pooled by habitat type (either artificial or natural) and reared to adults for morphological species identification as described below. Written informed consent was obtained from the head of each household prior to mosquito collection. To increase the probability of detecting *An. stephensi*, adaptive sampling was employed [44, 45]. Thus, 50% of the households for adult collection were replaced randomly in subsequent collection rounds.

#### Household exposure to *An. stephensi*

**Fig. 3:**
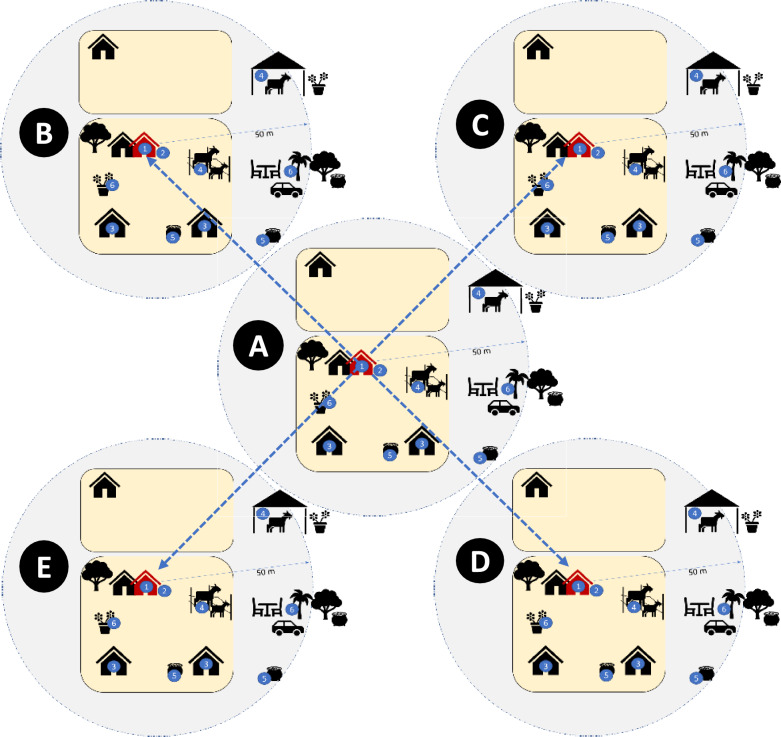
Diagrammatic representation of household selection for studying exposure to *An. stephensi*. Large shaded circles indicate the selected households together with additional structures within a 50-m radius for entomological collection. Shaded circle designated with** A** indicates the first selected household (in red) located toward the center of the town. Shaded circles designated with** B**–**E** indicate 4 additional households that were selected by moving 100 m (blue dotted line) in four directions from the first selected household. The remaining 45 households were selected by repeating this approach 9 times. The numbers in the blue circle indicate different types of structures within a 50-m radius of the selected household for adult or immature-stage mosquito collection

Four urban centers where *An*. *stephensi* was detected were selected for more detailed study: Awash Sebat Kilo (Afar Regional State), Danan (Somali Regional State), Metehara (Oromia Regional State) and Jiga (Amhara Regional State). At each selected site, adult mosquitoes were collected from 50 randomly selected households and their surroundings in December 2022 and February 2023. Using a map of urban centers, a household located toward the center of the town was selected first, and then four additional households were selected (at approximately a distance of 100 m, with each of these four dwellings located in a different direction from the first household). This approach was repeated 9 times, to select the remaining 45 households systematically (Fig. 3). Adult *Anopheles* collections were conducted both indoors and outdoors from the selected households using Centers for Disease Control and Prevention light traps (CDC LTs) and from any structures serving as potential resting places within the household’s property compound using a Prokopack aspirator (John W. Hock Company, Gainesville, FL, USA).

### Adult mosquito collections

Trained field workers collected adult mosquitoes from 06:00 to 08:00 h and from 17:30 to 19:00 h with Prokopack aspirators. Indoor resting mosquito collections were conducted along the walls and ceilings of houses and under or behind household furniture. Outdoor collections were made from vegetation, tree trunks or walls of water containers located outside of the selected structures. The collections were made for 15–30 min in each structure, with the time scaled to the number and size of the structures/areas. The head of the household was requested to avoid activities that could repel mosquitoes on the night before collection, such as smoking inside the house, using repellents or spraying aerosol insecticides [46, 47].

Host-seeking adult mosquitoes were collected both indoors and outdoors using CDC LTs. Indoor traps were set near a sleeping space at the foot edge next to an existing bed-net, and corresponding outdoor traps were set within 5–10 m of each household selected for the indoor collection. Traps were set approximately 1.5 m from the ground, both indoors and outdoors. At approximately 18:00 h, the battery was connected to run the trap, and the following morning, at 06:30 h, the trap was removed. The collection cup was tied off securely prior to the trap being switched off and then labeled with the associated household code, whether an indoor/outdoor collection and with the date of collection before being removed from the trap and the mosquitoes retrieved.

### Immature-stage mosquito collection and rearing

Aquatic habitats within a 50-m radius of the selected households were surveyed for immature stages, with the collections made using dippers and pipettes [48]. The collected immature-stage mosquitoes were subsequently transported to temporary field insectaries in plastic jars where they were transferred into enamel trays containing water from their respective aquatic habitat and larval food; the trays were check each day for pupation. Pupae were collected into beakers containing water from the respective aquatic habitat and placed in rearing cages with cotton balls soaked in a 10% sugar solution at the top of the cages. When all the pupae emerged, the beakers were removed from the rearing cages.

### Morphological identification and preservation of mosquito samples

All wild-caught adults and those reared from immature stages were sorted by genus and sex after anesthetization with 70% alcohol. Only female *Anopheles* and *Aedes* mosquitoes were counted and recorded. Female mosquitoes belonging to the genus *Anopheles* were identified morphologically to the species level. *Anopheles* mosquitoes with speckled legs, with maxillary palpus with two apical pale bands very broad, with speckling on palpus segment three and with second main dark area on wing vein one with two pale interruptions were identified as *An. stephensi* [49]. Wild-caught female adult *Anopheles* mosquitoes were sorted based on their abdominal status as freshly fed or unfed, then preserved individually in a 1.5-ml Eppendorf tube with holes at the top and sealed in Ziplock bags containing silica gel. The immature-stage (larvae and pupae) collections were pooled by the household and preserved in absolute ethanol for molecular identification of *An. stephensi*.

### Molecular procedures

The head and thorax of mosquitoes were separated from the abdomen for molecular analyses carried out at the Armauer Hansen Research Institute (AHRI) and Jimma University (JU) Tropical Infectious Diseases Research Center (TIDRC) laboratories. The bisected parts or pooled immature-stage materials were homogenized using a BioSpec BeadBeater apparatus (Bead Homogenizer 96 Microplate; Biospec Products, Bartlesville, USA) in 150 µl of molecular-grade water with 0.2 mg of beads (diameter: 1.0 mm; composition: zirconia/silica; BioSpec Products) at 3800 rpm for 20 s. DNA was extracted from 50- and 100-µl samples of homogenate according to the manufacturer's instructions (Qiagen GmbH, Hilden, Germany) and used for the detection of *Plasmodium* infection (head-thorax), blood meal analysis (abdomen), molecular species identification (immature stages) and confirmation of morphological identification (adult stage).

A PCR endpoint assay targeting the internal transcribed spacer 2 region (ITS2) was performed for species identification of *An. stephensi* as described elsewhere [50]. Briefly, the PCR was performed using the ST-F (5’CGTATCTTTCCTCGCATCCA3’), U5.8S-F (5’ATCACTCGGCTCATGGATCG3’) and UD2-R (5’GCACTATCAAGCAACACGACT3’) universal primers in a total reaction mixture of 25 µl containing 1 µl of DNA template, 0.25 µM each of ST-F and U5.8S-F primers, 0.37 µM of the UD2-R primer, 0.25 µM of dNTPs, 1.5 µM of Mg and 0.5 µM of Taq polymerase). PCR cycling conditions were: 95 °C for 30 s; 30 cycles of 95 °C for 30 s, 55 °C for 30 s and 68 °C for 7 min; with a final extension step at 68 °C for 7 min. The identification of specimens as *An. stephensi* was based on the visualization of a 438-bp band following gel electrophoresis of the PCR products.

### Detection of* Plasmodium* infection

*Plasmodium* parasites in wild-caught *Anopheles* mosquitoes were identified either by PCR (AHRI laboratory) or enzyme-linked immunosorbent assay (ELISA) (Jimma University TIDRC laboratory). The PCR used to detect *Plasmodium* parasites targeted the cytochrome c oxidase subunit 1 (COXI) mitochondrial gene, and the presence of the parasites was based on the visualization of a 540-bp region following gel electrophoresis of the PCR products, as described elsewhere [51, 52]. Briefly, the PCR was performed in a total reaction volume of 25 µl containing 3 µl of DNA template from the head and thorax, primers (0.25 µM), dNTPs (0.2 µM), Mg (1.5 µM) and 1 unit of Taq polymerase. For those samples which tested PCR-positive for COXI, a nested PCR was run targeting the small 18S subunit of *P. falciparum* and *P. vivax* [53]. The first amplification reaction (nested-1) was performed in a final reaction volume of 25 µl containing 3 µl of DNA template, and the second reaction was performed by using a 5 µl amplicon of nested-1. The presence of *P. falciparum* and *P. vivax* was confirmed upon visualization of 205- and 120-bp bands, respectively, following electrophoresis of the PCR products on 1% agarose gel.

To detect *Plasmodium* via ELISA, the head-thorax of each mosquito was separated from the abdomen and ground in blocking buffer containing IGEPAL CA-630 (Sigma-Aldrich, St. Louis, MO, USA) in a 1.5-ml grinding tube. Antibodies against the circumsporozoite protein (CSP) of *P. falciparum*, *P. vivax-*210 (Pv-210) and *P. vivax-*247 (Pv-247) were detected using a sandwich CSP-ELISA [54, 55]. More specifically, 50 μl of species-specific capture monoclonal antibody (mAb) was added to each well of a micro-ELISA plate. The binding sites were blocked by adding 200 µl of blocking buffer and incubating the plate for 1 h at room temperature. Then, 50 μl of mosquito homogenate or control sample was added to the respective labeled wells. The plates were subsequently covered and incubated for 2 h at room temperature, following which 50 μl of peroxidase-linked mAb were then added to the wells and the plates incubated for 1 h at room temperature. Finally, 100 µl of peroxidase substrate solution (ABTS [2,2’-azino-bis 3-ethylbenzothiazoline-6-sulfonic acid]; Kirkegaard and Perry Laboratories [KPL], Gaithersburg, MD, USA) was added and the plate incubated for 30 min for *P. falciparum* and 60 min for *P. vivax*. Between the addition of each reagent or sample and the incubation, the wells were shaken and washed. Absorbance was read at 405–411 nm using an ELISA reader (model ELX800; BioTek, Winooski, VT, USA) after 30 and 60 min of incubation for *P. falciparum* and *P. vivax,* respectively. Samples with a value higher than twofold of the mean absorbance value of the negative controls were considered to be positive for *Plasmodium* parasites.

### Detection of blood meal sources

The blood meal source of wild-caught, freshly blood-fed female *Anopheles* mosquitoes was analyzed by PCR (AHRI laboratory) or ELISA (Jimma University TIDRC laboratory). For the PCR, the extracted DNA from the abdominal region of female *Anopheles* was analyzed by a multiplex PCR assay as previously described [56]. The PCR assay was performed in a reaction volume of 25 µl containing universal vertebrate-specific and species-specific primers for pigs, humans, goats, dogs and cows [0.2 μM of each primer], 1× GoTAQ (Promega, Madison, WI, USA), 1.5 mM MgCl_2_, 0.2 mM dNTPs and 13.85 µl of molecular-grade water. The PCR amplification conditions were: 95 °C for 5 min; 40 cycles of 95 °C for 60 s, 57 °C for 60 s and 72 °C for 60 s; with a final extension of 72 °C for 7 min. The PCR products were confirmed by gel electrophoresis.

For the ELISA, the abdomens of freshly fed female *Anopheles* mosquitoes were homogenized in phosphate-buffered saline (PBS) in a 1.5-ml grinding tube following a standard protocol [55]. More specifically, 100 μl of homogenized sample or control was loaded added into a well of an ELISA plate and the plate incubated for 2 h at room temperature, following which the wells were washed 3 times with 200 µl of PBS-Tween 20 solution. Then, 50 µl of host-specific peroxidase-labeled mAb of human, bovine and goat (Sigma-Aldrich) was added and incubated for 1 h in the dark, followed by 3 washes with 200 µl of PBS–Tween 20, with the plate shaken 5 times with each wash. Finally, 100 µl of ABTS was added to each well as the substrate for the peroxidase enzyme, and the mixture was incubated for 30 min. The plates were observed visually, and their optical density was read at 405–414 nm using an ELISA reader (model ELX800; BioTek). Samples with a value higher than twofold the mean absorbance value of the negative controls were considered to be positive for *Plasmodium* parasites.

### Data management and statistical analysis

The data were collected using tablets with data forms developed in REDCap [57, 58] and uploaded to the AHRI data server on a daily basis. Data were downloaded and cleaned using Microsoft Excel (Microsoft Corp., Redmond, WA, USA), and Microsoft Excel and Stata software release 14 (StataCorp LLC, College Station, TX, USA) were used for analysis. Only *Anopheles* mosquito catches identified to species level were included in the statistical analyses. Site positivity proportion was determined by dividing the number of sites at which *Anopheles* species were identified in at least one round of entomological surveys by the number of sites surveyed. To estimate catches per method of collection, method-specific catches of species were divided by the total number of that species caught. Relative abundance of *Anopheles* species was investigated per site as catches of specific species divided by the total adult-stage collections per site. The mean number of catches per trap was determined by considering the number of traps in all collection rounds. Household exposure was defined as the presence of either immature or adult stages of *An. stephensi* in a 50-m radius of surveyed households. The level of household-exposure was estimated as the fraction of the surveyed households per number of households detected with *An. stephensi*. Mixed blood meal sources were included in the nominator to determine the blood meal indices. Sporozoite rate was determined as the fraction of *Anopheles* species tested per that species detected with *Plasmodium* parasite by PCR and ELISA.

## Results

### *Anopheles* mosquito fauna and abundance

**Fig. 4:**
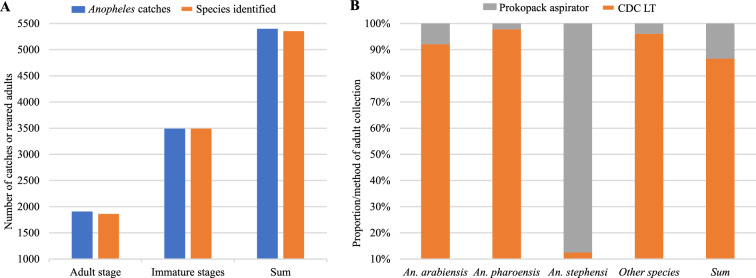
*Anopheles* mosquito catches. **A** total number of *Anopheles* mosquitoes collected and morphologically identified to species level. **B** proportion of adult *Anopheles* catches according to method of collection. CDC LT, U.S. Centers for Disease Control and Prevention light traps

Among the study sites, 96.2% (25/26) were positive for *Anopheles* mosquitoes, including *An. stephensi*, and 99.2% (5353/5398) of the total catches were identified to species level. Of the total adult *Anopheles* catches, 86.6% (1612/1862) were collected by CDC LTs, and 13.4% (250/1862) were collected by Prokopack aspirators (Fig. 4), with 79.5% of adult catches identified as *An. arabeinsis*, 7.7% as *An. stephensi* and 7.4% as *An. pharoensis*. *Anopheles coustani, An. tenebrosus, An. fuenstus and An. rufipes* accounted for only 2.5%, 1.8%, 1.1% and 0.1% of adult catches, respectively*. Anopheles stephensi* was predominant in four sites of the 26 surveyed (Babile, Kebri Dehar, Danan and Modjo), with 77.3–100% relative abundance. Among the immature-stage collections, *An. stephensi* was predominant in five sites of the 26 surveyed (Babile, Kebri Dehar, Danan, Dubti, and Modjo), with 75–100% relative abundance. *Anopheles arabiensis* was the predominant species across most sites (20/26) (Table 1). In two study sites, *Anopheles* mosquitoes were either not found (Kebri Beyah) or not identified morphologically (Yabelo) due to damage during collections.

**Table 1:** Distribution and abundance of *Anopheles* mosquitoes across study urban centers, Ethiopia, from surveys performed in 2021–2023

Site	*Anopheles* species	*N*	Relative abundance of species (%)	Mean number of* Anopheles *mosquitoes per trap	Method of collection					
					CDC LT			PRO		
					*n*	%	Mean number of* Anopheles *mosquitoes per trap	*n*	%	Mean number of* Anopheles *mosquitoes per trap
Afambo	*An. arabiensis*	634	81.9	7.93	598	94.3	7.48	36	5.7	0.9
	*An. pharoensis*	106	13.7	1.33	105	99.1	1.31	1	0.9	0.03
	*An. tenebrosus*	34	4.4	0.43	34	100	0.43			
Akaki	*An. arabiensis*	22	100	0.28	21	95.5	0.26	1	4.5	0.03
Arba Minch	*An. arabiensis*	62	96.9	0.78	62	100	0.78			
	*An. pharoensis*	2	3.1	0.03	2	100	0.03			
Asendabo	*An. arabiensis*	22	78.6	0.28	10	45.5	0.13	12	54.5	0.3
	*An. fuenstus*	1	3.6	0.01	1	100	0.01			
	*An. coustani*	5	17.9	0.06	3	60	0.04	2	40	0.05
Assosa	*An. arabiensis*	8	50	0.1	8	100	0.1			
	*An. coustani*	6	37.5	0.08	6	100	0.08			
	*An. pharoensis*	1	6.3	0.01	1	100	0.01			
	*An. stephensi*	1	6.3	0.01	1	100	0.01			
Ataye	*An. arabiensis*	28	80	0.35	24	85.7	0.3	4	14.3	0.1
	*An. fuenstus*	3	8.6	0.04	3	100	0.04			
	*An. coustani*	4	11.4	0.05	4	100	0.05			
Babile	*An. stephensi*	3	75	0.04				3	100	0.08
	*An. rufipes*	1	25	0.01	1	100	0.01			
Bambasi	*An. arabiensis*	3	50	0.04	3	100	0.04			
	*An. coustani*	2	33.3	0.03	2	100	0.03			
	*An. pharoensis*	1	16.7	0.01	1	100	0.01			
Bonga	*An. arabiensis*	8	100	0.1	7	87.5	0.09	1	12.5	0.03
Danan	*An. stephensi*	15	100	0.19	4	26.7	0.05	11	73.3	0.28
Dembiya	*An. arabiensis*	29	100	0.36	18	62.1	0.23	11	37.9	0.28
Dilla	*An. arabiensis*	16	94.1	0.2	16	100	0.2			
	*An. pharoensis*	1	5.9	0.01	1	100	0.01			
Dubti	*An. arabiensis*	47	70.1	0.59	38	80.9	0.48	9	19.1	0.23
	*An. coustani*	2	3	0.03	2	100	0.03			
	*An. pharoensis*	11	16.4	0.14	11	100	0.14			
	*An. stephensi*	7	10.4	0.09	7	100	0.09			
Gambella	*An. arabiensis*	9	42.9	0.11	9	100	0.11			
	*An. fuenstus*	4	19	0.05	4	100	0.05			
	*An. pharoensis*	8	38.1	0.1	8	100	0.1			
Harbu	*An. arabiensis*	132	97.8	1.65	120	90.9	1.5	12	9.1	0.3
	*An. fuenstus*	1	0.7	0.01	1	100	0.01			
	*An. coustani*	1	0.7	0.01				1	100	0.03
	*An. pharoensis*	1	0.7	0.01	1	100	0.01			
Jiga	*An. arabiensis*	87	76.3	1.09	84	96.6	1.05	3	3.4	0.08
	*An. coustani*	17	14.9	0.21	17	100	0.21			
	*An. pharoensis*	3	2.6	0.04	1	33.3	0.01	2	66.7	0.05
	*An. stephensi*	7	6.1	0.09	7	100	0.09			
Kebri Dehar	*An. stephensi*	20	95.2	0.25	5	25	0.06	15	75	0.38
	*An. pharoensis*	1	4.8	0.01	1	100	0.01			
Kombolcha	*An. arabiensis*	30	78.9	0.38	26	86.7	0.33	4	13.3	0.1
	*An. fuenstus*	7	18.4	0.09	6	85.7	0.08	1	14.3	0.03
	*An. coustani*	1	2.6	0.01	1	100	0.01			
Metamma	*An. arabiensis*	17	100	0.21	12	70.6	0.15	5	29.4	0.13
Modjo	*An. stephensi*	91	97.8	1.14				91	100	2.28
	*An. arabiensis*	2	2.2	0.03	2	100	0.03			
Omorate	*An. arabiensis*	38	97.4	0.48	34	89.5	0.43	4	10.5	0.1
	*An. pharoensis*	1	2.6	0.01	1	100	0.01			
Salamago	*An. arabiensis*	230	100	2.88	226	98.3	2.83	4	1.7	0.1
Shewa Robit	*An. arabiensis*	22	81.5	0.28	16	72.7	0.2	6	27.3	0.15
	*An. fuenstus*	4	14.8	0.05	4	100	0.05			
	*An. coustani*	1	3.7	0.01	1	100	0.01			
Woreta	*An. arabiensis*	34	81	0.43	29	85.3	0.36	5	14.7	0.13
	*An. coustani*	7	16.7	0.09	7	100	0.09			
	*An. pharoensis*	1	2.4	0.01	1	100	0.01			
Total	*An. arabiensis*	1480	79.5	0.47	1363	92.1	0.66	117	7.9	0.11
	*An. coustani*	46	2.5	0.01	43	93.5	0.02	3	6.5	
	*An. fuenstus*	20	1.1	0.01	19	95	0.01	1	5	
	*An. pharoensis*	137	7.4	0.04	134	97.8	0.06	3	2.2	
	*An. rufipes*	1	0.1		1	100				
	*An. stephensi*	144	7.7	0.05	24	16.7	0.01	120	83.3	0.12
	*An. tenebrosus*	34	1.8	0.01	34	100	0.02			

* CDC LT* U.S. Centers for disease control and prevention light traps, *N*, total number of catches, *n* number of catches per method,* %* proportion of catches per method,* PRO* Prokopack aspirator

### Spatiotemporal distribution of *An. stephensi*

In four rounds of entomological surveys, 30.8% (8/26) of the sites were positive for *An. stephensi*, immature and/or adult stages. Both adult and immature stages of *An. stephensi* were detected at six of these positive sites (Babile, Danan, Dubti, Jiga, Kebri Dehar, and Modjo), while at the remaining two sites either adults (Assosa) or immature stages (Ataye) were detected (Table 2). In four of the *An. stephensi*-positive sites, adult or immature stages were recorded every round. The mean number of wild-caught adult *An. stephensi* per trap was 0.15 (CDC LT: 0.04 per trap night; Prokopack aspirator: 0.38 per collection) (Additional file 1: Table S2). *Anopheles stephensi* larvae were collected from a range of artificial and natural aquatic habitats.

**Table 2:** Spatiotemporal distribution of *Anopheles stephensi* across study urban centers, Ethiopia, 2021–2023

*Anopheles stephensi*-positive sites	Collection rounds^a^ and stages caught							
	Round 1		Round 2		Round 3		Round 4	
	Adult	Immature	Adult	Immature	Adult	Immature	Adult	Immature
Assosa	−	−	−	−	+	−	−	−
Ataye	−	+	−	−	−	−	−	−
Babile	−	+^A^	+	−	−	−	−	−
Danan	+	+^A^	+	+^A^	+	+^A^	+	+
Dubti	−	+^A^	+	+^AN^	+	+^A^	+	+^A^
Jiga	+	−	+	+^N^	+	−	+	+^N^
Kebri Dehar	+	+^A^	+	+^A^	+	+^A^	+	+^A^
Modjo	−	+^A^	+	+^A^	−	+^A^	−	−

Key: −, Negative for both stages; +, positive for wild-caught adults; +^N^, positive for immature stages in natural habitat; +^A^, positive for immature stages in artificial habitat; +^AN^: positive for immature stages in artificial and natural habitat

^a^Round number and associated months of mosquito collections. 1: January-May; 2: June-November; 3: October-November; 4: December-January

### Household exposure to* An. stephensi*

During additional extensive entomological sampling in and around households, *An. stephensi* was detected in three of the four urban centers, namely Danan, Awash Sebat Kilo and Metehara. Among the 50 surveyed households, 30% (15/50) were positive for *An. stephensi* in Danan. The household positivity rates were 26% (13/50) and 18% (9/50) in Awash Sebat Kilo and Metehara, respectively. *Anopheles stephensi* was not detected in any of the sampled households in Jiga during the sampling period (Table 3).

**Table 3:** Household exposure rates for immature, adult and both stages of *Anopheles stephensi* across four urban centers, Ethiopia, 2023

Study sites	Houses surveyed (*n*)	Wild-caught adult-stage mosquitoes				Immature-stage collection				Both mosquito stages	Total HHP,* n* (%)
		*N*	CDC LT	PRO	HHP,* n* (%)	Aquatic habitats	AHP,* n* (%)	*N*	HHP,* n* (%)	HHP,* n* (%)	
Awash S.K.	50	9		9	3 (6)	Birka	2 (67)	94	10 (20)		13(26)
						Plastic drum	8 (80)	158			
Danan	50	21	3	18	9 (18)	Birka	5 (83)	378	5 (10)	1 (2)	15(30)
						Plastic tank	1 (100)	20			
Jiga	50	29	29			Ground water		3			
Metehara	50	26	10	16	2 (4)	Birka	5 (83)	564	6 (12)	1 (2)	9(18)
						Plastic drum	2 (100)	46			

*AHP* Aquatic habitat positivity rate for *An. stephensi*,* CDC LT* U.S. Centers for disease control and prevention light traps,* HHP* household positivity rate for *An. stephensi*, *N*, total number of* Anopheles* caught, *n* number of habitats or houses positive for *An. stephensi, PRO* Prokopack aspirator

### Species identification by molecular methods

A total of 80 field-caught adult mosquitoes identified as *An. stephensi* based on morphological characteristics and 28 pooled immature-stage mosquito samples were screened using molecular methods to confirm species identity. Of the 80 adult samples identified morphologically as *An. stephensi*, the molecular assays confirmed the identity as *An. stephensi* in 78 samples; in the remaining two samples, the products were either not amplified or were found to be non-*An. stephensi*. Among the pooled immature-stage mosquito samples, 82.1% (23/28 pools) were confirmed to contain *An. stephensi*.

### Detection of blood meal sources

Blood meals were analyzed in 784 *Anopheles* mosquitoes, of which 386 samples were assayed by multiplex PCR (Additional file 1: Fig. S1) and 398 specimens were analyzed using an ELISA. Among *Anopheles* mosquitoes identified with a blood meal source, 76.9% (50/65) of *An. stephensi* and 56.2% (181/322) of *An. arabiensis* were found with single host blood*.* The blood meal indices for *An. stephensi* were 69.2% for bovines (BBI), 32.3% for ovines (OBI) and 24.6% for humans (HBI), including the mixed blood meal sources (Table 4); for *An. arabiensis,* the blood meal indices were 65.4% for bovines, 46.7% for ovines and 35.8% for humans.

**Table 4:** Determination of blood meal sources of wild-caught adult *Anopheles* mosquitoes collected across urban centers in Ethiopia using multiplex PCR and enzyme-linked immunosorbent assay, 2021–2023

Site	*Anopheles* species	*N*	HBI		BBI		OBI		CBI		UN,* n* (%)
			S, *n* (%)	T, *n* (%)	S, *n* (%)	T, *n* (%)	S, *n* (%)	T, *n* (%)	S,* n* (%)	T, *n* (%)	
Assayed using multiplex PCR											
Afambo	*An. arabiensis*	211	15 (7.1)	28 (26.2)	56 (26.5)	72 (34.1)	10 (4.7)	20 (9.5)	5 (2.4)	8 (3.8)	104 (49.3)
	*An. pharoensis*	22	2 (9.1)	4 (40)	4 (18.2)	6 (27.3)	1 (4.5)	2 (9.1)		1 (4.5)	12 (54.5)
	*An. tenebrosus*	6	1 (16.7)	1 (20)	3 (50)	3 (50)	1 (16.7)	1 (16.7)			1 (16.7)
Akaki	*An. arabiensis*	13			2 (15.4)	3 (23.1)		1 (7.7)			10 (76.9)
Arba Minch	*An. arabiensis*	1		1 (100)						1 (100)	
Awash S. K	*An. stephensi*	7				1 (14.3)	3 (42.9)	4 (57.1)			3 (42.9)
Danan	*An. stephensi*	52	1 (1.9)	9 (23.7)	25 (48.1)	33 (63.5)	4 (7.7)	4 (7.7)			14 (26.9)
Kebri Dehar	*An. stephensi*	22	2 (9.1)	2 (14.3)	6 (27.3)	6 (27.3)	6 (27.3)	6 (27.3)			8 (36.4)
Metehara	*An. stephensi*	3	1 (33.3)	2 (66.7)			1 (33.3)	2 (66.7)			
Omorate	*An. arabiensis*	18	3 (16.7)	4 (66.7)	2 (11.1)	2 (11.1)		1 (5.6)			12 (66.7)
Salamago	*An. arabiensis*	29	9 (31)	15 (78.9)	1 (3.4)	3 (10.3)			3 (10.3)	7 (24.1)	10 (34.5)
*Assayed by ELISA*											
Asendabo	*An. arabiensis*	14				7 (50)	2 (14.3)	9 (64.3)			5 (35.7)
	*An. funestus*	1				1 (100)		1 (100)			
	*An. coustani*	2				1 (50)	1 (50)	2 (100)			
Assosa	*An. arabiensis*	5		1 (100)		1 (20)		1 (20)			4 (80)
	*An. coustani*	6				2 (33.3)		2 (33.3)			4 (66.7)
Ataye	*An. arabiensis*	9			2 (22.2)	5 (55.6)	3 (33.3)	6 (66.7)			1 (11.1)
	*An. coustani*	2				2 (100)		2 (100)			
Bambasi	*An. coustani*	1				1 (100)		1 (100)			
Bonga	*An. arabiensis*	3				1 (33.3)	1 (33.3)	2 (66.7)			1 (33.3)
Dembiya	*An. arabiensis*	14		2 (20)	2 (14.3)	7 (50)	3 (21.4)	8 (57.1)			4 (28.6)
Dubti	*An. arabiensis*	42		6 (35.3)	2 (4.8)	7 (16.7)	8 (19)	13 (31)			25 (59.5)
	*An. pharoensis*	10				1 (10)	1 (10)	2 (20)			8 (80)
Gambella	*An. arabiensis*	4			1 (25)	1 (25)					3 (75)
	*An. funestus*	4	1 (25)	1 (100)							3 (75)
Harbu	*An. arabiensis*	112	10 (8.9)	16 (30.8)	10 (8.9)	34 (30.4)	6 (5.4)	32 (28.6)			60 (53.6)
	*An. pharoensis*	1			1 (100)	1 (100)					
	*An. coustani*	1	1 (100)	1 (100)							
Jiga	*An. arabiensis*	77	7 (9.1)	29 (50.9)	7 (9.1)	43 (55.8)	5 (6.5)	38 (49.4)			20 (26)
	*An. pharoensis*	2	1 (50)	1 (50)		1 (50)		1 (50)			
	*An. coustani*	13	1 (7.7)	6 (66.7)		7 (53.8)	1 (7.7)	7 (53.8)			4 (30.8)
	*An. stephensi*	7	1 (14.3)	3 (50)		5 (71.4)		5 (71.4)			1 (14.3)
Kombolcha	*An. arabiensis*	15	1 (6.7)	4 (44.4)		7 (46.7)	1 (6.7)	8 (53.3)			6 (40)
	*An. funestus*	5				2 (40)		2 (40)			3 (60)
Metemma	*An. arabiensis*	9	1 (11.1)	4 (100)		2 (22.2)		1 (11.1)			5 (55.6)
Shewa Robit	*An. arabiensis*	8			1 (12.5)	8 (100)		7 (87.5)			
	*An. funestus*	4			1 (25)	4 (100)		3 (75)			
Woreta	*An. arabiensis*	9	1 (11.1)	5 (71.4)	1 (11.1)	6 (66.7)		3 (33.3)			2 (22.2)
	*An. coustani*	6	2 (33.3)	2 (100)							4 (66.7)
Total	*An. arabiensis*	595	47(7.9)	115 (19.3)	87 (14.6)	209 (35.1)	39 (6.6)	150 (25.2)	8 (1.3)	16 (2.7)	273 (45.9)
	*An. coustani*	31	4 (12.9)	9 (29)		13 (41.9)	2 (6.5)	14 (45.2)			12 (38.7)
	*An. funestus*	14	1 (7.1)	1 (7.1)	1 (7.1)	7 (50)		6 (42.9)			6 (42.9)
	*An. pharoensis*	39	3 (7.7)	5 (12.8)	5 (12.8)	9 (23.1)	2 (5.1)	5 (12.8)		1 (2.6)	23 (59)
	*An. stephensi*	99	5 (5.1)	16 (16.2)	31 (31.3)	45 (45.5)	14 (14.1)	21 (21.2)			34 (34.3)
	*An. tenebrosus*	6	1 (16.7)	1 (16.7)	3 (50)	3 (50)	1 (16.7)	1 (16.7)			1 (16.7)

*BBI* Bovine blood meal index,* CBI* canine blood meal index,* ELISA* enzyme-linked immunosorbent assay,* HBI* human blood meal index,* OBI* ovine blood meal index, *N* number of* Anopheles* tested,* n* number of samples with S, T and UN, respectively,* S* single blood meal source,* T* single + mixed blood meal source,* UN* unidentified blood meal source

### Detection of *Plasmodium *infection

Among the 1847 *Anopheles* mosquitoes examined for *Plasmodium* infection, 69.4% (1282/1847) and 30.6% (565/1847) were assessed using PCR (Additional file 1: Fig. S2) and ELISA, respectively (Table 5). None of the 197 samples identified as *An. stephensi* were found to be infected with *Plasmodium* parasites. Overall, the *Plasmodium* sporozoite rate of *An. arabiensis* was 4.3% (62/1434), of which 16.1% (10/62) were *P. falciparum* and 83.9% (52/62) were *P. vivax*. In *An. pharoensis*, the* Plasmodium* sporozoite rate was 6.7% (8/119), of which 12.5% (1/8) were *P. falciparum* and 87.5% (7/8) were *P. vivax*. Two other *Anopheles* mosquito species were detected with sporozoites of *P. vivax*: *An. coustani *(12.8%, 5/39) and *An. funestus* (11.5%, 3/26).

**Table 5:** Sporozoite rate in wild-caught adult *Anopheles* mosquitoes collected across study urban centers, Ethiopia, 2021–2023

*Anopheles* species	*Plasmodium* species^a^	Mosquito collection sites^b^												
		Afa	Aka	Arb	Awa	Bab	Dan	Dil	Dah	Met	Mod	Omo	Sal	Total
Assayed using multiplex PCR														
*An. stephensi*	T				9	3	56		20	3	91			179
	*PF, n (%)*													
	*PV, n (%)*													
*An. arabiensis*	T	614	21	61				16			2	34	219	967
	*PF, n (%)*	3 (0.5)										1 (2.9)	2 (0.9)	6 (0.6)
	*PV, n (%)*	2 (0.3)		1 (1.6)									2 (0.9)	5 (0.5)
*An. pharoensis*	T	98		2								1		101
	*PF, n (%)*	1 (1)												1 (1)
	*PV, n (%)*	3 (3.1)												3 (3)
*An. tenebrosus*	T	34												34
	*PF, n (%)*													
	*PV, n (%)*													
Assayed by CSP-ELISA														
		Ase	Ass	Ata	Bam	Bon	Dem	Dub	Gam	Har	Jig	Kom	Sho	Total
*An. stephensi*	T		1					7			7			15
	*PF, n (%)*													
	*PV, n (%)*													
*An. arabiensis*	T	24	7	31	4	9	30	55	9	183	89	18	8	467
	*PF, n (%)*	2 (8.3)					2(6.7)							4 (0.9)
	*PV, n (%)*	3 (12.5)	1 (14.3)	6 (19.4)	1 (25)	2 (22.2)	4 (13.3)	1 (1.8)	1 (11.1)	18 (9.8)	6 (6.7)	3(16.7)	1 (12.5)	47 (10.1)
*An. pharoensis*	T		1		1			11	1	1	3			18
	*PF, n (%)*													
	*PV, n (%)*				1 (100)			1 (9.1)	1 (100)		1 (33.3)			4 (22.2)
*An. funestus*	T	1		3					11			7	4	26
	*PF, n (%)*													
	*PV, n (%)*	1 (100)		1 (33.3)								1 (14.3)		3 (11.5)
*An. coustani*	T	5	6	4	3	1		2		1	16		1	39
	*PF, n (%)*													
	*PV, n (%)*			1 (25)		1 (100)		1 (50)			2 (12.5)			5 (12.8)

*CSP-ELISA* Circumsporozoite protein-enzyme-linked immunosorbent assay

^a^*Plasmodium* species:* PF P. falciparum*,* PV P. vivax*.* n*, Number of infected mosquitoes; T, number tested

^b^Afa, Afambo; Aka, Akaki; Arb, Arba Minch, Awa, Awash Sebat Kilo; Bab, Babile; Dan, Danan; Dil, Dilla; Dah, Kebri Dehar; Met, Metehara; Mod, Modjo; Omo, Omorate; Sal, Salamago; Ase, Asendabo; Ass, Assosa; Ata, Ataye; Bam, Bambasi; Bon, Bonga; Dem, Dembiya; Dub, Dubti; Gam, Gambella; Har, Harbu; Jig, Jiga; Kom, Kombolcha; Sho, Shewa Robit

## Discussion

The results of the present study add to the body of available evidence on the distribution and abundance of invasive *An. stephensi* in Ethiopia. Entomological surveillance in 26 urban centers between 2021 and 2023 revealed that *An. arabiensis* was the predominant* Anopheles* species in the catches, accounting for 79.5% of all collections, followed by *An. stephensi*, accounting for 7.7% of the total *Anopheles* catches. The relative abundance of adult *An. stephensi* was greater than that of *An. arabiensis* in Babile, Kebri Dehar, Danan and Modjo. Modjo is located along the main ground transportation route or corridor that connects Ethiopia to Djibouti [36]. Generally, adult *An. stephensi* collections were low (mean: 0.15 catches/trap), and most of the immature-stage collections were from artificial aquatic habitats.

Since the first detection of *An. stephensi* in eastern Ethiopia in 2016, new positive sites have been identified in subsequent surveys [36, 37, 59]. In line with these findings, we detected *An. stephensi* in western Ethiopia (Assosa), in an area bordering Sudan, which might indicate the continued spread of this species. We also found *An. stephensi* at all previously reported sites, as well as at new sites along its purported invasion route. However, the site positivity of *An. stephensi* was relatively low (8/26 sampled sites) compared to that reported in previous studies. A study in 2020 that covered 10 sites in eastern Ethiopia reported the presence of *An. stephensi* at all sites [36]. Similarly, sampling at 21 sites between 2018 and 2020 revealed 61.9% positivity for *An. stephensi* [37]. Another study conducted by the PMI Vector Link Ethiopia project showed the presence of *An. stephensi* in 16 urban settings, of which nine (56.3%) sites were newly positive for *An. stephensi* [60]. One explanation for the differences in results might be the selection of study sites; most of the collection points in these previous studies were purposefully chosen to detect *An. stephensi*, while the current study followed substantial random steps in the selection of study sites and collection points within sites. Our approach has the advantage of providing unbiased distribution estimates, but it does reduce the probability of detection. In addition, some of our study sites were located far from major transportation corridors [61], which are considered as the main invasion routes.

In line with the findings of other studies [16, 36], we also noted that *An. stephensi* was more readily detectable as immature stages than as adults in most of the positive sites. The highest proportion of *An. stephensi* collections (85.7%) was obtained as immature stages (larvae and pupae) in aquatic habitats. A range of aquatic habitats were positive; for example, in Dubti, immature stages of *An. stephensi* were detected in both artificial habitats (water tanks, barrels, buckets, tires) and natural habitats (ponds, streams, swamps and marshes). It has been reported that *An. stephensi* can breed in various aquatic habitats with differing physicochemical characteristics, such as salinity and turbidity [62]. In Modjo, Danan, Kebri Dehar and Babile, immature stages of *An. stephensi* were detected only in artificial habitats. This variation highlights how larval source management of *An. stephensi,* which has been recommended by the WHO [63] and is being implemented by PMI VectorLink and others, will be more complex than simply targeting container habitats.

In the current study, *An. arabiensis* was the most abundant species at 20/26 sites, which is in line with the findings of other studies showing that this species is still the predominant malaria vector in different eco-epidemiological settings of Ethiopia [20, 25, 64]. Even though *An. arabiensis* is considered less adapted to urban ecology [65], our findings suggest that it is likely to be the primary malaria vector in urban centers in Ethiopia. The other *Anopheles* species collected in this study were *An. pharoensis, An. coustani, An. funestus, An. tenebrosus* and *An. rufipes*, which together accounted for 12.8% of the total adult *Anopheles* catches. Of these five species, *An. pharoensis* and *An. funestus* have been reported to be secondary or suspected malaria vectors in Ethiopia [66]. We detected *An. pharoensis* infected with *P. falciparum* or *P. vivax* at one and five of our study sites, respectively, while *An. coustani* and *An. funestus* were detected with *P. vivax* sporozoites across four and three of the study sites, respectively*.*

The level of household exposure to *An. stephensi* was heterogeneous across the study sites, with household positivity for both (adults and immature) stages ranging from 18% in Metehara to 30% in Danan. The level of household exposure to adult *An. stephensi* was highest in the region where this species was first reported as an invasive species (Danan) and lower in more central parts of the country (Awash Sebat Kilo and Metehara). A similar trend was observed for household exposure to the immature stages of *An. stephensi*. These results could be due to well-established populations of *An. stephensi* in areas where it was first reported since its invasion.

Our findings reveal that *An. stephensi* prefers non-human vertebrate hosts for their blood meal. The most prevalent blood meal among *An. stephensi* detected with sources of blood was cattle (69.2%), followed by goats (32.3%). These findings are consistent with results previously reported in Ethiopia [16, 37] and India [67], which showed that most *An. stephensi* fed on livestock. In the present study, one-third of *An. stephensi* fed on unidentified blood meal sources, which might be due to a lack of host antibodies or primers for blood meal analysis. It is noteworthy that at some of the study sites, especially in eastern Ethiopia, the most readily available animals were camels and that these sites were where most of the tested *An. stephensi* were collected. Despite the relatively high non-human vertebrate host blood meal indices, 24.6% of *An. stephensi* were found with human blood, including mixed blood meal sources. The blood meal source of vectors might be affected by multiple factors, including host availability and proximity, possibly explaining why 76.9% of the *An. stephensi* and 56.2% of *An. arabiensis* fed on a single blood meal source of either an animal or a human host.

Of the 197 screened *An. stephensi*, none were detected with *Plasmodium* parasites. These findings are similar to those of another study in which none of the tested *An. stephensi* was positive for *Plasmodium* [36]. However, a study conducted in 2019 in Awash Sebat Kilo reported an infection rate of 2.8% for *P. vivax* and 1.4% for *P. falciparum*, based on analysis of homogenates of whole mosquitoes [16]. The authors of another study in which the heads and thoraxes was used to detect *Plasmodium* by ELISA reported that the sporozoite rate was 0.5% in Dire Dawa and 0.3% in Kebri Dehar for *P. vivax* [37]. The most recent study, from Dire Dawa, implicated *An. stephensi* in an outbreak and detected a *P. falciparum* sporozoite rate of 1.2% [38]. It should be noted that our study might be limited in its ability to elucidate *An. stephensi* sporozoite infection, as a large proportion of the adult catches (91/146) were from a single aquatic site using the Prokopack aspirator.

There are a number of limitations to our study. The limited number of *An. stephensi* adults caught indicates the need for further studies to investigate more efficient trapping methods. We employed both PCR and ELISA for detecting blood meal sources and *Plasmodium* infection in *Anopheles* mosquitoes, which might limit the direct comparability of our findings across study sites. The collection rounds did not directly coincide with the malaria transmission seasons in Ethiopia, and some of the sites were excluded due to civil unrest.

## Conclusions

Our findings reveal an expansion of *An. stephensi* into a new western geographical range and its transition to predominant species status in some areas where it was first detected. These results highlight the need for enhanced entomological surveillance with efficient traps to determine the bionomics and relative contribution of *An. stephensi* for malaria transmission in the region. In the meantime, the plan set forth to limit the spread and contain *An. stephensi* establishment should be put into action.

### Supplementary Information

**Additional file 1: Table S1**. Study sites in Ethiopia, 2021–2023. **Table S2:** Occurrence and abundance of *An. stephensi* across eight positive urban centers by round and stage of collection, Ethiopia, 2021–2023. **Figure S1.** Multiplex PCR for blood meal source detection in freshly fed wild-caught female *Anopheles* mosquitoes. **Figure S2.** Gel images of COXI and nested PCR for detecting *Plasmodium* infection.

## Data Availability

The datasets reported herein were shared with stakeholders, including the Ministry of Health and the WHO. All the data from the CEASE project will be made publicly available upon completion of the study.

## Abbreviations

AHRI: Armauer Hansen Research Institute
BBI: Bovine blood index
CDC LTs: U.S. Centers for Disease Control and Prevention light traps
COXI: Cytochrome c oxidase subunit 1
CSP: Circumsporozoite protein
ELISA: Enzyme-linked immunosorbent assay
HBI: Human blood index
ITS2: Internal transcribed spacer 2 region
mAb: Monoclonal antibody
OBI: Ovine blood index
PBS: Phosphate-buffered saline
REDCap: Research Electronic Data Capture
