# Supplementary material for: Spatiotemporal distribution and bionomics of Anopheles stephensi in different eco-epidemiological settings in Ethiopia
Source: Parasit Vectors. 2024 Mar 31;17:166. doi: 10.1186/s13071-024-06243-3 (PMC10983662; doi:10.1186/s13071-024-06243-3)
Supplement: Supplementary file 1 — Additional file 1: Table S1. Study sites in Ethiopia, 2021–2023. Table S2: Occurrence and abundance of An. stephensi across eight positive urban centers by round and stage of collection, Ethiopia, 2021–2023. Figure S1. Multiplex PCR for blood meal source detection in freshly fed wild-caught female Anopheles mosquitoes. Figure S2. Gel images of COXI and nested PCR for detecting Plasmodium infection. [file 13071_2024_6243_MOESM1_ESM.docx]

## Additional file 1

**Table S1: Study sites in Ethiopia, 2021-2023.**

| Study site | Region | Altitude (masl) | Latitude | Longitude |
| --- | --- | --- | --- | --- |
| Akaki | Addis Ababa | 2350 | 8.861858 | 38.786833 |
| Afambo | Afar | 356 | 11.518777 | 41.587164 |
| Dubti | Afar | 367 | 11.733201 | 41.082054 |
| Ataye | Amhara | 1458 | 10.346294 | 39.955860 |
| Woreta | Amhara | 1830 | 11.894478 | 37.676753 |
| Dembiya | Amhara | 1832 | 12.421638 | 37.324065 |
| Harbu | Amhara | 1479 | 10.923906 | 39.780413 |
| Jiga | Amhara | 1630 | 10.671131 | 37.372587 |
| Kombolcha | Amhara | 1798 | 11.078880 | 39.734166 |
| Metemma | Amhara | 713 | 12.948649 | 36.163057 |
| Shoa Robit | Amhara | 1278 | 10.002236 | 39.894093 |
| Bambasi | Benishangul Gumuz | 1468 | 9.748421 | 34.739811 |
| Assosa town | Benishangul Gumuz | 1572 | 10.055242 | 34.549032 |
| Gambella | Gambella | 445 | 8.232059 | 34.579175 |
| Babile | Oromia | 1648 | 9.228092 | 42.334374 |
| Modjo | Oromia | 1788 | 8.585457 | 39.133908 |
| Yabelo | Oromia | 1857 | 4.809572 | 36.054702 |
| Asendabo | Oromia | 1740 | 7.762966 | 37.226017 |
| Arba Minch | SNNPR | 1285 | 6.055508 | 37.556389 |
| Dilla | SNNPR | 1570 | 6.399215 | 38.303658 |
| Omorate | SNNPR | 371 | 4.809102 | 36.053733 |
| Salamago | SNNPR | 509 | 6.221887 | 36.129891 |
| Danan | Somalia | 435 | 6.513210 | 43.492950 |
| Kebri Beyah | Somalia | 1609 | 9.100243 | 43.174230 |
| Kebri Dehar | Somalia | 532 | 6.726662 | 44.276125 |
| Bonga | SWE | 1600 | 7.291270 | 36.234960 |

*Keys: masl: meters above sea level, SNNPR: Southern Nations, Nationalities, and Peoples' Region, SWE: South West Ethiopia Peoples' Region*

**Table S2:** Occurrence and abundance of *An. stephensi* across eight positive urban centers by round and stage of collection, Ethiopia, 2021-2023.

| Positive sites | Mosquito stages | Habitat type/Collection method | | Rounds of mosquito collection | | | | Total/site | |
| --- | --- | --- | --- | --- | --- | --- | --- | --- | --- |
|  |  |  | I | | II | III | IV | |  |
| Assosa | Reared adults | Artificial (n) | - | | - | - | - | | - |
|  |  | Natural (n) | - | | - | - | - | | - |
|  | Wild caught adults | CDC, n(ct) | - | | - | 1 (0.05) | - | | 1 (0.01) |
|  |  | PRO, n(ct) | - | | - | - | - | | - |
| Ataye | Reared adults | Artificial (n) | - | | - | - | - | | - |
|  |  | Natural (n) | 1 | | - | - | - | | 1 |
|  | Wild caught adults | CDC, n(ct) | - | | - | - | - | | - |
|  |  | PRO, n(ct) | - | | - | - | - | | - |
| Babile | Reared adults | Artificial (n) | 20 | | - | - | - | | 20 |
|  |  | Natural (n) | - | | - | - | - | | - |
|  | Wild caught adults | CDC, n(ct) | - | | - | - | - | | - |
|  |  | PRO, n(ct) | - | | 3 (0.3) | - | - | | 3 (0.08) |
| Danan | Reared adults | Artificial (n) | 9 | | 58 | 67 | 16 | | 150 |
|  |  | Natural (n) | - | | - | - | - | | - |
|  | Wild caught adults | CDC, n(ct) | 2 (0.1) | | 1 (0.05) | - | 1 (0.05) | | 4 (0.05) |
|  |  | PRO, n(ct) | 4 (0.4) | | 1 (0.1) | 1 (0.1) | 5 (0.5) | | 11 (0.28) |
| Dubti | Reared adults | Artificial (n) | 5 | | 226 | 126 | 28 | | 385 |
|  |  | Natural (n) | - | | 13 | - | - | | 13 |
|  | Wild caught adults | CDC, n(ct) | - | | 6 (0.3) | 1 (0.05) | - | | 7 (0.09) |
|  |  | PRO, n(ct) | - | | - | - | - | | - |
| Jiga | Reared adults | Artificial (n) | - | | - | - | - | | - |
|  |  | Natural (n) | - | | 2 | - | 5 | | 7 |
|  | Wild caught adults | CDC, n(ct) | 1 (0.05) | | 1 (0.05) | 2 (0.1) | 3 (0.15) | | 7 (0.09) |
|  |  | PRO, n(ct) | - | | - | - | - | | - |
| Kabri Dehar | Reared adults | Artificial (n) | 36 | | 40 | 20 | 20 | | 116 |
|  |  | Natural (n) | - | | - | - | - | | - |
|  | Wild caught adults | CDC, n(ct) | 2 (0.1) | | - | 2 (0.1) | 1 (0.05) | | 5 (0.06) |
|  |  | PRO, n(ct) | 3 (0.3) | | 2 (0.2) | 8 (0.8) | 2 (0.2) | | 15 (0.38) |
| Modjo | Reared adults | Artificial (n) | 66 | | 70 | 46 | - | | 182 |
|  |  | Natural (n) | - | | - | - | - | | - |
|  | Wild caught adults | CDC, n(ct) | - | | - | - | - | | - |
|  |  | PRO, n(ct) | - | | 91 (9.1) | - | - | | 91 (2.28) |
| Total/round | Reared adults | Artificial (n) | 136 | | 394 | 259 | 64 | | 853 |
|  |  | Natural (n) | 1 | | 15 | - | 5 | | 21 |
|  | Wild caught adults | CDC, n(ct) | 5 (0.03) | | 8 (0.05) | 6 (0.04) | 5 (0.03) | | 24 (0.04) |
|  |  | PRO, n(ct) | 7 (0.09) | | 97 (1.21) | 9 (0.11) | 7 (0.09) | | 120 (0.38) |

*Keys: n: number of catches, CDC: Centre for disease control and prevention light trap, PRO: Prokopack aspirator: ct: mean number of An. stephensi catch/trap, and I (Jan-May), II (Jun-Nov), III (Oct-Nov) and IV (Dec-Jan): rounds and months of mosquito collection.*


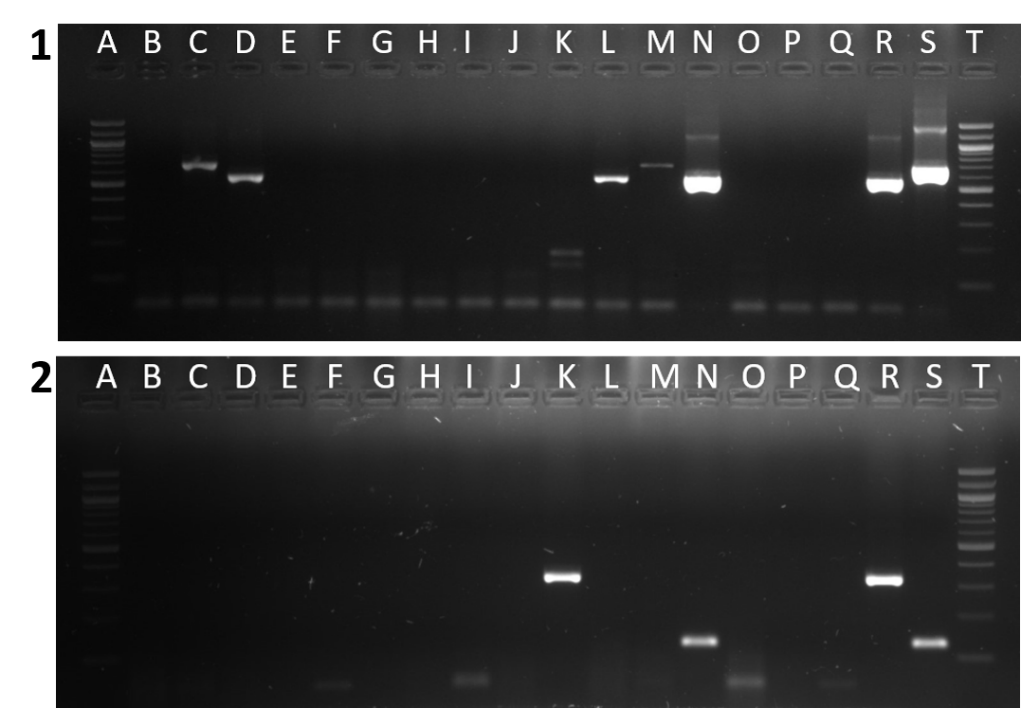


***Figure S1:*** *Multiplex PCR for blood meal source detection in freshly fed wild-caught female Anopheles mosquitoes. (1) A and T are 100 bp ladders; Q, negative control; R, cow positive control; S, dog positive control; B-P, DNA samples with C and M, positive for dog blood, whereas D, L and N are positive for cow blood. (2) A and T were ladders 100 bp, Q, extraction negative control; R, human positive control; S, goat positive control; B-P DNA samples with K positive for human blood whereas N positive for goat blood.*


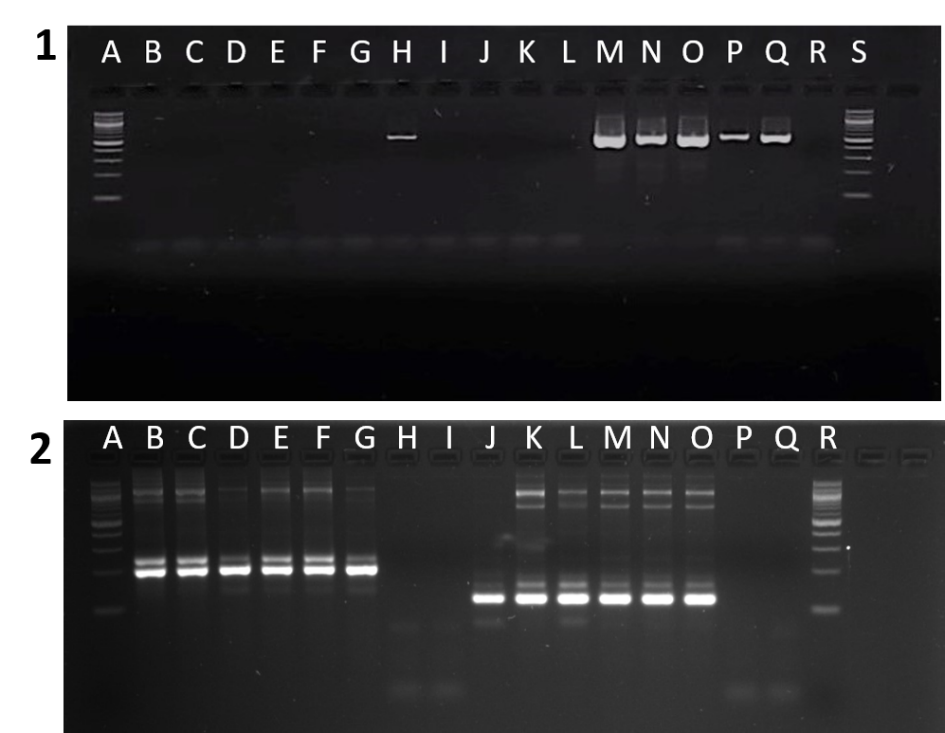


***Figure S2:*** *Gel images of COX1 and nested PCR for detecting Plasmodium infection. (1) COX1 PCR; A and S are 100 bp ladders; M-Q are extraction positive controls (membrane-fed mosquitoes on gametocyte-positive P. vivax clinical patients with an infectivity rate of 89%); R, extraction negative control (mosquitoes head and thorax from reared adults from immature collection); and B-L, samples with H positive for Plasmodium. (2) From nested PCR optimization, A and R are 100 bp ladders, B-G is a P. falciparum positive control, H-I and P-Q are negative controls and J-O are P. vivax positive controls.*
